# Use of an Integrated Research-Practice Partnership to Improve Outcomes of a Community-Based Strength-Training Program for Older Adults: Reach and Effect of Lifelong Improvements through Fitness Together (LIFT)

**DOI:** 10.3390/ijerph15020237

**Published:** 2018-01-31

**Authors:** Meghan L. Wilson, Thomas E. Strayer, Rebecca Davis, Samantha M. Harden

**Affiliations:** 1 Department of Human Nutrition, Foods, and Exercise, Virginia Polytechnic Institute and State University, Blacksburg, VA 24061, USA; harden.samantha@vt.edu; 2 Translational Biology, Medicine, and Health, Virginia Polytechnic Institute and State University, Roanoke, VA 24016, USA; strayert@vt.edu; 3 Virginia Cooperative Extension, Virginia Polytechnic Institute and State University, Blacksburg, VA 24061, USA; rdavis58@vt.edu

**Keywords:** physical activity, functional fitness, aging in place, evidence-based program, cooperative extension

## Abstract

Only 17% of older adults meet the recommendations for two days of full body strength training that is associated with improved functional fitness; reduced risk of falls; and reduced morbidity and mortality rates. Community-based interventions are recommended as they provide supportive infrastructure to reach older adults and impact strength training behaviors. Scalability and sustainability of these interventions is directly linked with setting-level buy-in. Adapting an intervention through an integrated research–practice partnership may improve individual and setting-level outcomes. The purpose of this study was to evaluate the initial reach and effect of a locally adapted, health educator-led strength-training intervention; Lifelong Improvements through Fitness Together (LIFT). LIFT was compared to an evidence-based exercise program, Stay Strong; Stay Healthy (SSSH). Intervention dose and mode were the same for LIFT and SSSH, but LIFT included behavioral change strategies. Older adult functional fitness was assessed before and after the 8-week strength training intervention. Health educators who delivered LIFT and SSSH were able to reach 80 and 33 participants, respectively. Participants in LIFT were able to significantly improve in all functional fitness measures whereas SSSH participants were only able to significantly improve in 5 of the 7 functional fitness measures. In conclusion, this study provides preliminary evidence that the locally adapted program reached more individuals and had improvements in functional fitness.

## 1. Introduction

Older adults (≥65 years of age) represent the fastest growing and most inactive subset of individuals in the United States [1]. Although two days of full-body strength-training recommendations were included in the physical activity guidelines a decade ago [2], only 17% of older adults meet the recommendations for strength-training [3]. This low rate of compliance with strength-training recommendations is a public health concern.

Physiologically, older adults lose muscle mass and strength with increased age and physical inactivity [4,5]. Muscle weakness hinders older adults’ ability to continue their daily routine and remain functionally fit, or perform everyday activities safely and independently without tiredness or weakness [6]. Individuals with weakened quadriceps, hamstrings, or hip extensors muscles have reported slower walking speed and velocity [4], issues climbing stairs, and problems with standing from a seated position [7]. Similarly, upper body muscle weakness is associated with decreased ability to perform daily household chores such as folding laundry, gardening, and carrying grocery bags [8]. Older adults who engage in strength-training exercises are able to maintain or improve muscle mass and strength [7] along with remaining functionally fit as they age [6].

Functional fitness can be objectively measured through a previously validated seven-item test [6] and should be a key outcome of interest associated with strength-training interventions. This measure was initially reported as ‘user-friendly’ by those administering the test and ‘enjoyable’ by those participating in the test [6]; however, it is often not used in its entirety [9,10]. For example, two community-based programs—EnhanceFitness [9] and Strong-for-Life Program [10]—used an adapted version of the Rikli and Jones protocol in practice to determine the effect of the intervention on functional fitness.

While there are a number of efficacious and effective community-based strength-training programs for older adults [11], there is little evidence indicating that the interventions are successfully translated into practice-based settings beyond the length of the intervention. This lack of translation may be a result of a top-down dissemination approach where an academic researcher produces a program and suggests that a community should deliver it [12]. This development and delivery approach often disregards community-specific needs, values, and abilities to deliver research-developed programs [13]. For example, a community may lack the resources (e.g., location, equipment, etc.) or staff with expertise to deliver evidence-based strength-training programs [14]. An evidence-based program will have a much greater public health impact if they are integrated into a practice-based setting and delivered consistently beyond the length of the intervention [15].

Rather than a top down approach, an integrated research–practice partnership (IRPP) between communities and research academics may speed program translation into a practice-based setting [16,17]. Through an IRPP, academic researchers and invested communities can work collaboratively to develop and deliver an intervention that adheres to the evidence-based principles of the intervention *and* complements the setting in which it will be delivered [18]. This approach encourages equal buy-in from both the academic research team and the community delivering the program [19]. Communities with buy-in of an evidence-based program may deem it more suitable for delivery if it has been adapted in accordance with the mission, values, and resources of the practice-based setting [20,21,22] and therefore will ultimately reach more of the target population and have a greater effect [19] on functional fitness. An IRPP approach may bridge the gap of translating effective, evidence-based programs into community-based settings beyond the length of the intervention [16].

One delivery system that has the capability of delivering evidence-based programs to a large proportion of older adults is the national cooperative extension system (herein: Extension) [23]. For years, Extension-delivered programs focused primarily on educating individuals about food and nutrition with the incorporation of physical activity as a less important aspect of educational programs [24]. Since physical activity objectives were added to the National Institute of Food and Agriculture strategic plan in 2014, efforts to incorporate physical activity programming in Extension are inchoate.

With this strategic goal in mind, county-based health educators of Extension serve as appropriate facilitators of physical activity programming. As one example of this potential impact, over 30 states deliver a version of an exercise-science based Strong Women, Strong Bones [25] program for older adults whereas over 20 states deliver a statewide walking program [26]. Related to the former, in a recent dissemination trial of Strong Women, Strong Bones, of the 881 people trained on the program, 43% were Extension professionals [25]. Most of the states currently delivering the program in practice adhere to the core elements of the exercise science of Strong Women, Strong Bones, but few programs have shared their adaptations to the program nor their ongoing reach or effect in the community. One investigator has shared branding adaptations and the continued impact on fall prevention of the exercise science principles of the program [27]. This program was renamed Stay Strong, Stay Healthy (SSSH) to attract male participants; the outcome paper reports that SSSH was delivered by 34 Extension nutrition and health specialists to a total of 808 older adult participants. SSSH participants were able to significantly improve in all measures of the Rikli and Jones functional fitness assessment protocol from pre-program to post-program [27].

In order to bring the exercise science principles of Stay Strong, Stay Healthy to a new population and setting, adaptations [28] were required to fit the mission, values, and resources of the state system [16,18]. Additionally, when adaptations are made, there are concerns for internal and external validity of the intervention (i.e., does the adapted program work with this new population, in this new setting). This provides a critical need to describe the IRPP process and the outcomes that led to program adaptations. The degree to which the adapted program can reach community members, improve objectively measured functional fitness outcomes, and build capacity among county-based health educators to continue to support the program within their state must be evaluated. 

Taken together, (1) community delivered evidence-based programs are necessary to reach and impact older adult physical activity behaviors [29]; (2) these behaviors should be objectively measured to give veracity to the community-based intervention [30]; (3) focusing on the reach and representativeness of the target audience is essential for improved generalizability; and (4) in order to improve system level perceptions of fit, a participatory approach is recommended [17,31]. The purpose of this feasibility study was to evaluate the potential reach and effect of a locally adapted version of Strong Women, Strong Bones. It is hypothesized that adapting an intervention to meet the needs of the delivery setting may increase the reach and effect of evidence-based strength training programs for older adults [16].

## 2. Materials and Methods

### 2.1. Program Design

In 2015, an IRPP among county-based health educators and the new exercise specialist of Virginia Cooperative Extension (VCE) was formed. Through a needs assessment, it was identified that physical activity promotion among aging adults was a high priority for the IRPP and the communities they served. One health educator on the IRPP was trained on the Strong Women, Strong Bones [25] program when she worked in a different state Extension system. One critique of the Strong Women, Strong Bones program for VCE was that the program name was not as inclusive for male participants. In looking to the peer reviewed literature, members of the IRPP found Missouri Extension’s Stay Strong, Stay Healthy program [27]. This program was open to and actively recruited male participants, and, therefore, was the targeted program rather than the original Strong Women, Strong Bones intervention.

However, additional adaptations of Stay Strong, Stay Healthy were needed to meet the mission and values of VCE. Members of the IRPP proposed to incorporate behavior change strategies of Activity for the Ages [21], fruit and vegetable consumption component, and self-monitoring for aerobic activity outside of the strength-training sessions. The resultant program was called Lifelong Improvements through Fitness Together (LIFT) [20]. More information about the process and the Adaptome-based [28] changes of SSSH to LIFT are reported elsewhere [20] however, these adaptations are also summarized in Table 1.

### 2.2. Interventions

LIFT and SSSH were intended for older (≤65), sedentary men and women and delivered by Extension county-based health educators two times per week for eight weeks, totaling 16 sessions. Each session was designed to last an hour and consisted of strength-training, balance, and flexibility exercises that equaled eight total exercises. The exercises were as follows: wide-leg squat, standing leg curl, seated knee extension, side hip raise, biceps curl, overhead press, seated row, and toe stand. All sessions began with an active warm-up, followed by the eight exercises, and finished with a cool-down. The cool-down stretches were as follows: a hamstring and calf stretch, chest and arm stretch, and an upper backstretch (see Supplementary Figure S1 for a diagram of the strength training exercises and cool-down stretches). The exercise structure and contact time for LIFT and SSSH were the same. The health educators implemented behavior change strategies for LIFT participants during the entire program but particularly during warm up and cool down (see Figure S1, Tables S1 and S2). For example, during the warm up of session 4, there was discussion of motivators and barriers of physical activity and within the cool down of session 5, participants were encouraged to set goals for the following week.

### 2.3. Research Design

This study was a pragmatic, randomized, controlled feasibility trial. Health educators of VCE (*N* = 52) were invited to attend in-person training on physical activity promotion interventions. The physical activity training was hosted by the lead and senior authors and included training on (1) the primary evidence-based principles of obtaining physical activity behavior change, (2) strength-training exercises (proper form, cues to deliver, safe speed, etc.), (3) the importance of evaluation tools (surveys, functional fitness assessment, and demographic information, and (4) personal experience and experiential practice in leading groups through strength-training exercises (all training materials are available upon request).

Before a full launch of LIFT across the state of Virginia, it was essential to test the feasibility of the adaptations and build the evidence-base of the program. Health educators who attended the 2015 training and were still interested in translating LIFT into their communities (*n* = 13) were invited to attend a three-hour, in-person information session in January of 2016. Specific to the January 2016 training, health educators were (1) re-familiarized with the adapted program, LIFT and (2) given repeat demonstrations of the eight strength-training exercises and functional fitness assessments. Following the training, nine of the thirteen health educators of Extension agreed to be randomized to deliver LIFT or SSSH within their community as part of the feasibility study during the months of May through August 2016.

### 2.4. Older Adult Participants

Extension health educators recruited older adults (≤65) through email, community bulletin boards, newspaper articles, phone calls, etc. LIFT participants (*N* = 80) and SSSH participants (*N* = 33) were required to complete the Physical Activity Readiness Questionnaire (PAR-Q) [32]. In any case that a participant did not pass the PAR-Q, they must have obtained written support from their primary care physician before participating in LIFT or SSSH. All eligible individuals who agreed to participate were given a detailed description of the study, contact information of the principal investigators, and provided informed consent. Functional fitness assessments were conducted before and after the study along with a pre-program and post-program survey. Participants were encouraged to attend all 16 sessions.

All study procedures were completed in accordance with the approval of the Virginia Tech Institutional Review Board (#16-032). Health educators and older adults provided written informed consent prior to participation in this study. Participation in this study was voluntary and all data were kept confidential.

### 2.5. Measures

#### 2.5.1. Reach, Representativeness, and Retention

The pre-program survey assessed self-report, socio-demographics to gather data regarding the reach, or number, proportion, and representativeness of LIFT participants [33]. Reach data were calculated as a proportion of the total eligible population (inactive adults ages 65 and older in the state of Virginia based on the United States Census Bureau-Virginia) and the total sample enrolled in LIFT. Representativeness, or the degree to which the sample reflected the target population, was calculated based on the total eligible population for important demographic characteristics such as sex, race, and ethnicity.

Attendance was also recorded at the beginning of each program session to assess overall retention rate, or the proportion of participants who were present at follow-up. Present at follow-up was given a dichotomous variable (1 = completed and therefore retained, 0 = incomplete and therefore not retained) based on the participants’ completion of post-program survey or a post-program functional fitness test.

#### 2.5.2. Cohesion

The Physical Activity Group Environment Questionnaire (PAGE-Q) was previously validated in a group-based program for older adults to determine individuals’ perceptions of task and social integration as a measure of group cohesion [34]. There are four dimensions of cohesion: an individuals’ attraction to the group’s task (ATG-T), an individuals’ attraction to the group socially (ATG-S), the unity of the group around a task (i.e., exercise) (GI-T), and the unity of the group socially (GI-S) [35]. Individuals’ attractions to the group were assessed using items such as “I like the amount of physical activity I get in this program” (ATG-T) and “my group is an important social outlet for me” (ATG-S). The dimensions for group integration were assessed using items such as “our group works together to achieve our physical activity goals” (GI-T) and “our group spends time socializing with each other” (GI-S). For the purposes of this trial, three additional constructs were included to assess friendly competition, (e.g., “there is friendly competition within our group to stay as healthy as possible”), cooperation (e.g., “we all cooperate to help the program run smoothly”), and task- and social-based communication (e.g., “our group discusses the importance of regular physical activity” (task) and “people in my group talk about things that are happening in our lives” (social)). These additional measures were previously tested in a group dynamics program for racial and ethnic minority women [36]. There are three items associated with each construct with the exception of task-based communication. An additional item for task communication was added to address strength-training specifically (“members of our group talk about how often they should do strength training”). A 5-point Likert scale was used, in which 1 = strongly disagree, 3 = neither agree nor disagree, and 5 = strongly agree.

#### 2.5.3. Effectiveness

The previously validated and reliable functional fitness test by Rikli and Jones [6] was used to measure functional fitness outcomes of all participants. The functional fitness test measures upper and lower body strength, aerobic endurance, upper and lower body flexibility, and agility/dynamic balance [6]. For ease of administering the test, the 2-min step-test was used to measure aerobic endurance in place of the 6-min walk. The pre-program functional fitness test was conducted to establish a base-line physical activity assessment for each participant, while the post-program functional fitness test was conducted to determine maintenance or any changes in functional fitness capabilities following the 8-week strength-training (LIFT or SSSH) program. The health educator for each cohort obtained the functional fitness assessments. Practitioners collected data as it is more reflective of what would happen in practice to obtain outcome data. Secondly, the same health educator conducted assessments at pre- and post- measurement time points to ensure consistency.

To evaluate balance, a graded balance test [27] was done in conjunction with the pre- and post- functional fitness test. This assessment consists of six balance movements, each held for 10 s. If at any point a participant could not hold a position for 10 s, they did not move on to attempt the next balance movement. An overall composite balance score was calculated based on the number of balance movements the participants were able to complete. The graded balance test was used to be consistent with the SSSH protocol [27]. Going forward, a more widely used tool that has shown to be valid and reliable, the Berg Balance scale, will be used to assess older adult balance [37].

#### 2.5.4. System-Level Indicators

While not the primary purpose of this paper, adoption rate and system-level maintenance were also captured. Adoption data were operationalized as the proportion of eligible and interested health educators who agreed to deliver LIFT or SSSH as part of the feasibility study [20]. System-level maintenance data were operationalized as health educators’ intent to deliver the program in the future (1 = intend to deliver; 0 = do not intend to deliver) [20].

### 2.6. Statistical Analyses

Statistical analyses were performed using statistical analysis software (SPSS version 23.0 for Windows, IBM Corporation, Armonk, NY, USA). Conventional descriptive statistics were used to describe the demographics of LIFT and SSSH participants. Inferential statistics (chi-square tests) were used to estimate (due to sample size differences) if representativeness of the program was different from the target population. These methods were adapted from a previous peer-reviewed, older adult physical activity study [38]. Retention rate was reported as a percentage of participants who completed either a post-program functional fitness assessment or survey and those who did not complete either the post-program functional fitness assessment or survey.

Inferential statistics (*t*-tests) were used to detect change scores between pre- and post-functional fitness tests and PAGE-Q results. Chi-squared analyses were used to detect significant differences between pre- and post- balance test measures of those that completed each balance movement. Statistically significant differences were determined at a level of *p* ≤ 0.05.

## 3. Results

### 3.1. Reach, Representativeness, and Retention

**Figure 1:**
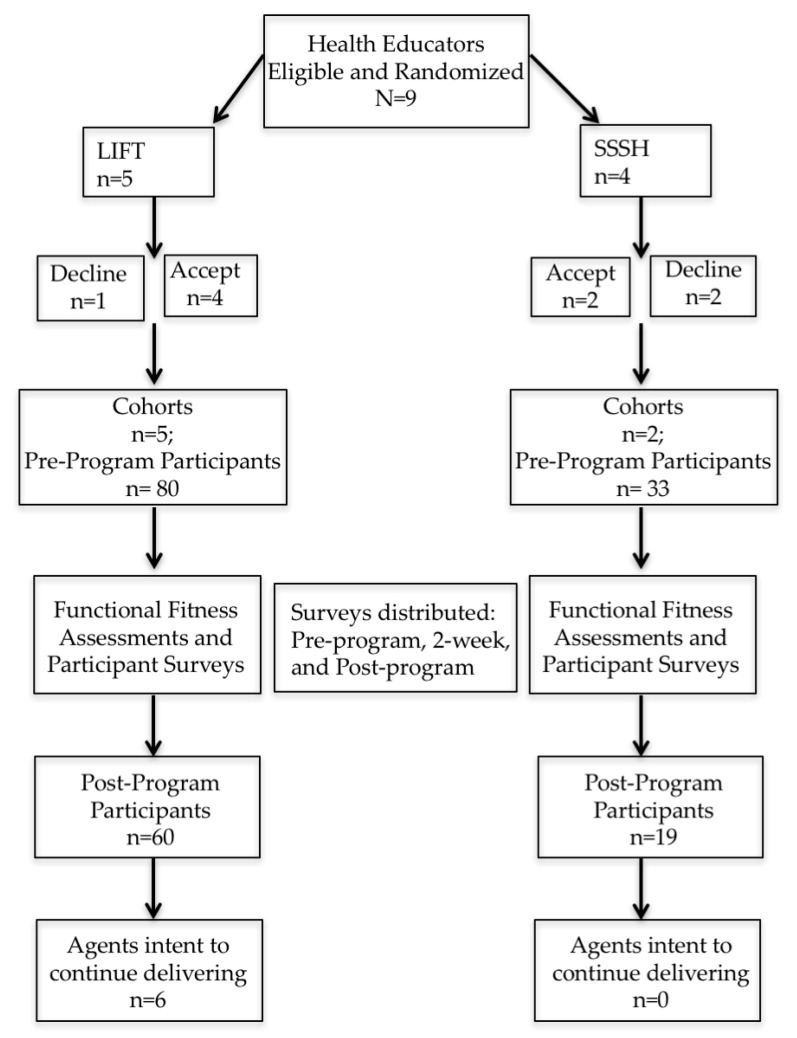
CONSORT diagram of the feasibility study design and outcomes based on the number of health educators who delivered LIFT or SSSH, the number of participants reached, assessment of functional fitness, participant retention rates, and number of health educators planning to deliver LIFT or SSSH in the future.

For the feasibility trial, four of the thirteen (31%) eligible health educators delivered LIFT to five cohorts of participants (one health educator delivered LIFT to two cohorts) and two (15%) eligible health educators delivered SSSH to two cohorts of participants. Health educators who delivered LIFT were able to reach a total of 80 older adult participants with an average starting class size of 20 (±11) individuals whereas those who delivered SSSH were able to reach 33 older adult participants with an average class size of 16.5 (±6) individuals (see Figure 1). Although the overall reach was significantly smaller compared to the number of eligible participants in the state of Virginia, our sample was representative of the total eligible population to participate based on ethnicity and race (see Table 2). However, the percentage of male participants in LIFT and SSSH was significantly smaller when compared to the overall eligible male population. However, SSSH was not representative in aspects of race (*p* < 0.01) and ethnicity (*p* < 0.01). Although not significantly different (*p* = 0.138), retention rates trended in the hypothesized direction in that LIFT retained 75% of the participants and SSSH retained 58% of the participants.

### 3.2. Effectiveness: Functional Fitness and Balance Tests

Functional fitness change scores (from baseline to post-program) were similar between intervention groups for the 30-s sit and stands, 2-min step test, lower and upper body flexibility, and the overall balance scores (see Table 3). However, LIFT participants were able to complete significantly more arm curls in 30 s (3.65 ± 6.03 versus 1.40 ± 6.37 arm curls) and significantly reduced their 8-foot-up-and-go time (−0.65 ± 1.31 s versus −0.06 ± 1.97 s) when compared to SSSH participants (Table 3).

### 3.3. System-Level Indicators

Of the 13 health educators who expressed interest in the feasibility study, 9 (70%) agreed to be randomized to deliver LIFT or SSSH. Five health educators were randomized to LIFT and four delivered or adopted within their community. Four health educators were randomized to SSSH and two adopted the program within their community.

One hundred percent of the health educators who delivered LIFT reported that they had intentions to deliver LIFT in the future. Of those who delivered SSSH, 0% confirmed their intentions to deliver SSSH in the future. However, the two health educators (100%) who delivered SSSH indicated their intention to deliver LIFT in the future. The health educators who delivered SSSH expressed their desire to incorporate the nutrition component and active facilitation of the social environment for their older adult participants.

## 4. Discussion

The primary purpose of this study was to determine the reach and effect (i.e., functional fitness) of an evidence-informed program that was adapted through an integrated research-practice partnership. This study reports a pragmatic approach for an evidence-based, health educator-led program, LIFT, to reach inactive older adults. Results of the study indicate that participation in an 8-week strength-training program can significantly improve the strength, flexibility, agility, dynamic balance, and aerobic endurance of older adults. These improvements in functional fitness enable older adults to live independently longer and more safely [39]. Although not significant, the group-based program, LIFT, had greater retention rates as well as delivery personnel intent to continue delivery.

There were more participants in the LIFT intervention arm when compared to the SSSH intervention arm. Although there were fewer health educators delivering this program, there were also more participants per health educator (20 participants per health educator in LIFT versus 16 per health educator in SSSH). Although the overall sample size may seem small compared to the overall older adult population in Virginia, the reach of LIFT was comparable to other physical activity interventions [40] and strength-training interventions [5]. With the exception of male versus female participants in the LIFT intervention arm, the data provide evidence that delivery through Extension was successful at reaching a representative sample of the overall target population. However, SSSH was not representative of the population in regard to race and ethnicity, which can be attributable to the geographical location that the SSSH intervention was delivered. Future recruitment efforts should aim to recruit more male participants. This may result in a strength-training program that has a more generalizable public health impact on physical activity behaviors of older adults.

In addition to concerns for reach and representativeness, the LIFT program was developed to specifically retain more people throughout the program. To align with this mission, LIFT was developed as a group dynamics-based intervention [21,41,42,43]. Group-based interventions have previously improved retention rates within exercise interventions [44,45] and the retention rate of LIFT was similar to other studies that reported on retention rates (~70%) [46,47]. Notably, the group dynamics literature has called for mediation analyses [48,49,50]. Specific to this study, full mediation [51] were not conducted as the first assumption was not met (i.e., retention within LIFT was not significantly greater than SSSH). Additionally, our sample did not show improvements for group cohesion from baseline to post-program for LIFT (see Table 4). However, both SSSH and LIFT participants initially reported high (≥3: neither agree nor disagree on the Likert scale) perceptions of group cohesion at baseline, leaving little room for improvement at the end of the sessions. High perceptions of group cohesion at baseline may have been a result of the participants being recruited from the same communities. Another limitation of this analysis was that there were very few participants who completed the PAGE-Q during the post-program survey.

The results of this study also indicated that older adult participants were able to significantly improve their functional fitness for strength, flexibility, aerobic endurance, agility, and dynamic balance. This evidence should not be surprising, as the literature suggests consistent participation in a strength-training program increases overall physical activity of older adult individuals [52]. However, many physical activity programs do not presently incorporate evidence-based behavior change strategies (e.g., goal-setting, group dynamics-based activities, and self-monitoring) [53,54,55]. Existing strength-training intervention studies have shown positive effects for individuals with improvements in strength, physical function, mobility, chronic illnesses, and most recently, a decrease in all-cause mortality [7,56,57]. However, the heterogeneous measures across these strength-training studies (e.g., self-report, direct observation, objective) leaves paucity in the literature with regard to improvements in functional fitness [29]. Therefore, future studies that objectively measure the outcomes of functional fitness for older adults are imperative.

This study also provided preliminary support that a theory-based intervention with behavior change strategies embedded within an exercise program resulted in improved functional fitness and retention rates. Further related, although self-report leisure-time physical activity was not assessed during this trial, three of the four health educators who delivered LIFT reported that their participants were either, a) inquiring about an advanced version of LIFT to participate in in the future, b) were hoping to join another cohort of LIFT at the end of the first eight weeks, and c) were continuing to meet at the end of eight weeks to continue the exercise routine one time weekly. Although these data are not measurable, they provide support that LIFT was able to impact physical activity behaviors, of a sup-sample of participants, beyond the length of the intervention. Future research, which is underway, is needed to explore long-term physical activity behaviors of older adults who have engaged in a strength-training program that targets behavioral change strategies (e.g., goal-setting, self-monitoring, self-efficacy, etc.).

This study did not come without limitations. First, there was a small sample size of health educators delivering LIFT to older adults. Our sample size can be justified as this was a pilot implementation study to ensure that LIFT was feasible and could be delivered as intended [20] within this community-based setting while improving the reach and effect for older adults. Twenty years of existing evidence supports the use of strength-training interventions to reach older adults and improve their physical activity behaviors [58] however, there is a dearth of transparent reporting on local-level adaptations and implementation to capture how adaptations may negatively or positively influence the effect of an intervention [28]. Second, health educators were not blind to randomization. This method for randomization was chosen as county-based health educators in the LIFT program needed to deliver the fruit/vegetable consumption portion and behavior change strategies whereas the SSSH health educators needed to adhere to the SSSH protocol. Results of this disappointment left potential for program drift [28]. Third, we were unable to gather sufficient 6-month, self-report physical activity follow-up data. Future studies should aim to collect follow-up data on physical activity behavior to ensure these interventions are resulting in long-term physical activity behavior change for older adults.

Taken together, LIFT improved all measures of functional fitness for older adults and retained those older adults throughout the entire intervention. Although literature supports the effectiveness of strength training interventions for older adults [11,59] it was necessary to conduct the feasibility trial to ensure that state-wide dissemination of LIFT would be evidence-based, not evidence-informed.

## 5. Conclusions

Delivery of LIFT by Cooperative Extension health educators had significant reach, representativeness, and improved effect for previously sedentary older adult participants. It is important to note that the four health educators who delivered LIFT and both health educators who delivered SSSH expressed their desire to continue or begin delivery of LIFT in the future. As the number of health educators delivering LIFT continues to increase, a greater number of older adult participants will be recruited to participate in LIFT. Reaching a greater proportion of the target population will result in an intervention that has a greater public health impact on the sedentary behaviors and functional fitness of older adults. To conclude, offering an open-access [60] community- and group dynamics-based strength-training intervention to older adults can successfully improve the reach and representativeness of an intervention while improving the functional fitness of older adults. These improvements may result in long-term physical activity behavior change of older adults, which may ultimately lead to older adults living independently longer (maintaining autonomy, free of chronic disease, etc.).

## Figures and Tables

**Table 1:** Intervention descriptions. Similarities and differences (adaptations made) between Stay Strong, Stay Healthy (SSSH) and Lifelong Improvements through Fitness Together (LIFT).

Component of Intervention	SSSH	LIFT
Duration	1-h sessions Participants encouraged to attend 2 times a week for 8 weeks	1-h sessions Participants encouraged to attend 2 times a week for 8 weeks Participants are encouraged to complete an additional thirty minutes of aerobic exercise outside of LIFT (e.g., walking, gardening, etc.)
Audience	Inactive, aging men and women	
Behavior change components	Not applicable	Observational learning Self-monitoring Self efficacy Group dynamics Relapse prevention Goal setting
Exercises	Active warm-up 8 core strength training exercises Cool down	
Group Dynamics	Not applicable	Group environment (e.g., small groups for interaction, group name) Group structure (e.g., group roles, group norms) Group processes (e.g., friendly competition, social support, group goal setting, etc.)
Goal	Increase muscle and bone density Decrease osteoporosis and frailty	Increase muscle and bone density Decrease osteoporosis and frailty Implement group dynamics-based behavior change strategies to increase program adherence and improve functional fitness of older adults

**Table 2:** Demographic information of total eligible population in Virginia (based on available census data) and demographic information of the sample participants enrolled in LIFT and SSSH.

Characteristics	Population ^a^ (*N* = 1,019,857.52)	LIFT (*n* = 80)	SSSH (*n* = 33)
Age, *M* (SD)	65+	72.55 (±11.06)	73.63 (±9.68)
BMI, *M* (SD)	27.8	30.56 (±6.23)	33.77 (±8.11)
Sex, %			
Male	44	14 *	9 *
Female	56	73 *	73 *
Not reported	-	13	18
Ethnicity, %			
Hispanic	3	3	0 *
Non-Hispanic	97	78	82 *
Not reported	-	19	18
Race, %			
White	79	56	27 *
Black	15	11	52 *
Other	6	1	3 *
Not reported	-	12	21

^a^ Data obtained from United States Census Bureau: Virginia. Inferential statistics (chi-squared tests) were used to determine significant differences between the representativeness of the total eligible population in Virginia compared to the total sample population of LIFT and SSSH (* *p* < 0.05).

**Table 3:** Pre- and post-program functional fitness change scores and composite balance change scores, by intervention.

Functional Fitness Assessment, *M* (SD)	Intervention	Baseline	Post-Program (ITT) ^a^	Change Scores
Sit and stands ^b^	LIFT	10.58 (±3.21)	13.07 (±5.14)	2.67 (±3.73) *
	SSSH	9.40 (±4.29)	10.71 (±3.22)	1.31 (±2.17) *
Arm curls ^b^	LIFT	13.89 (±4.0)	17.65(±6.22)	3.65 (±6.03) *
	SSSH	14.50 (±5.55)	15.9 (±4.3)	1.40 (±6.37)
2-min step test ^c^	LIFT	61.66 (±30.0)	77.5 (±30.0)	14.03 (±16.71) *
	SSSH	52.6 (±22.6)	72.4 (±32.3)	20.33 (±33.09) *
Lower body flexibility ^d^	LIFT	−1.74 (±3.86)	−0.0003 (±2.96)	1.77 (±2.97) *
	SSSH	−0.76 (±3.12)	0.68 (±3.05)	1.44 (±2.53) *
Upper body flexibility ^d^	LIFT	−5.05 (±4.93)	−4.2 (±5.51)	1.24 (±3.17) *
	SSSH	−6.05 (±5.69)	−4.8 (±4.06)	1.25 (±1.94) *
8-foot up-and-go ^e^	LIFT	7.68 (±3.84)	7.02 (±3.25)	−0.65 (±1.31) *
	SSSH	7.19(±2.92)	6.6 (±1.94)	−0.60 (±1.97)
Composite balance score ^f^	LIFT	2.44 (±1.3)	2.79 (±1.5)	0.35 (±1.18) *
	SSSH	2.00 (±1.0)	2.42 (±1.4)	0.42 (±0.99) *

^a^ ITT: Intention to Treat: A participant’s baseline score was included in the ITT analysis if they were not present for the post-program functional fitness assessment but did respond to the post-program survey. ^b^ Repititions completed in 30 s; ^c^ Number of steps taken in 2 min; ^d^ flexibility reported in inches; ^e^ time (in seconds); ^f^ total number of balance moves completed out of six. Paired *t*-test determined significant differences within program from baseline to post-program (ITT) (* *p* < 0.05).

**Table 4:** Pre- and post-program physical activity group environment questionnaire change scores on a 5-point Likert scale, by intervention.

Group Cohesion Dimension	Intervention	*N*	Baseline	*N*	Post Program	*N*	Change Score
Attraction to group: Task	LIFT	65	4.36 (±0.60)	40	4.38 (±0.72)	36	−0.19 (±0.48)
	SSSH	22	4.61 (±0.47)	17	4.51 (±0.99)	12	−0.06 (±1.07)
Attraction to group: Social	LIFT	65	3.67 (±0.81)	40	3.81(±0.84)	36	−0.12 (±0.69)
	SSSH	21	4.51 (±0.61)	17	4.24 (±1.04)	11	−0.06 (±1.14)
Group integration: Task	LIFT	65	3.89 (±0.63)	39	4.0 (±0.80)	35	−0.13 (±0.61)
	SSSH	22	4.58 (±0.51)	17	4.06 (±1.09)	12	−0.47 (±1.06) *
Group Integration: Social	LIFT	65	3.14 (±0.88)	35	−3.28 (±0.80)	32	0.04 (±0.94)
	SSSH	20	4.24 (±0.83)	17	3.84 (±1.12)	11	−0.03 (±1.32)
Cooperation	LIFT	64	4.17 (±0.56)	36	4.09 (±0.77)	33	−0.24 (±0.75)
	SSSH	21	4.38 (±0.60)	18	4.20 (±1.02)	12	−0.17 (±1.08)
Friendly Competition	LIFT	64	3.70 (±0.72)	35	3.53 (±0.86)	32	−0.23 (±0.76)
	SSSH	22	4.38(±0.73)	17	4.16 (±1.02)	12	−0.17 (±1.01)
Communication: Task	LIFT	64	3.68 (±0.80)	35	3.60 (±0.87)	32	−0.20 (±0.74)
	SSSH	22	4.14 (±0.71)	17	4.0 (±1.05)	12	−0.15 (±1.39) *
Communication: Social	LIFT	64	3.62 (±0.76)	35	3.74 (± 0.77)	32	0.02 (± 0.92)
	SSSH	22	4.29 (±0.75)	17	4.04 (±1.05)	12	−0.03 (±1.14)

*N*: the number of participants who responded to the survey and were included in the analyses (* *p* < 0.05).

## Supplementary Materials

The following are available online at http://www.mdpi.com/1660-4601/15/2/237/s1, Figure S1: LIFT and SSSH strength training exercises and cool-down stretches, Table S1: Session-by-session behavior change strategies and activities for LIFT, Table S2: Physical activity and fruit and vegetable tracking sheet.
