# Supplementary material for: Use of an Integrated Research-Practice Partnership to Improve Outcomes of a Community-Based Strength-Training Program for Older Adults: Reach and Effect of Lifelong Improvements through Fitness Together (LIFT)
Source: Int J Environ Res Public Health. 2018 Jan 31;15(2):237. doi: 10.3390/ijerph15020237 (PMC5858306; doi:10.3390/ijerph15020237)
Supplement: Supplementary file 1 [file ijerph-15-00237-s001.pdf]

International Journal of
Environmental Research and Public Health

MDPI

Supplementary Figure 1: LIFT and SSSH strength training exercises and cool-down stretches

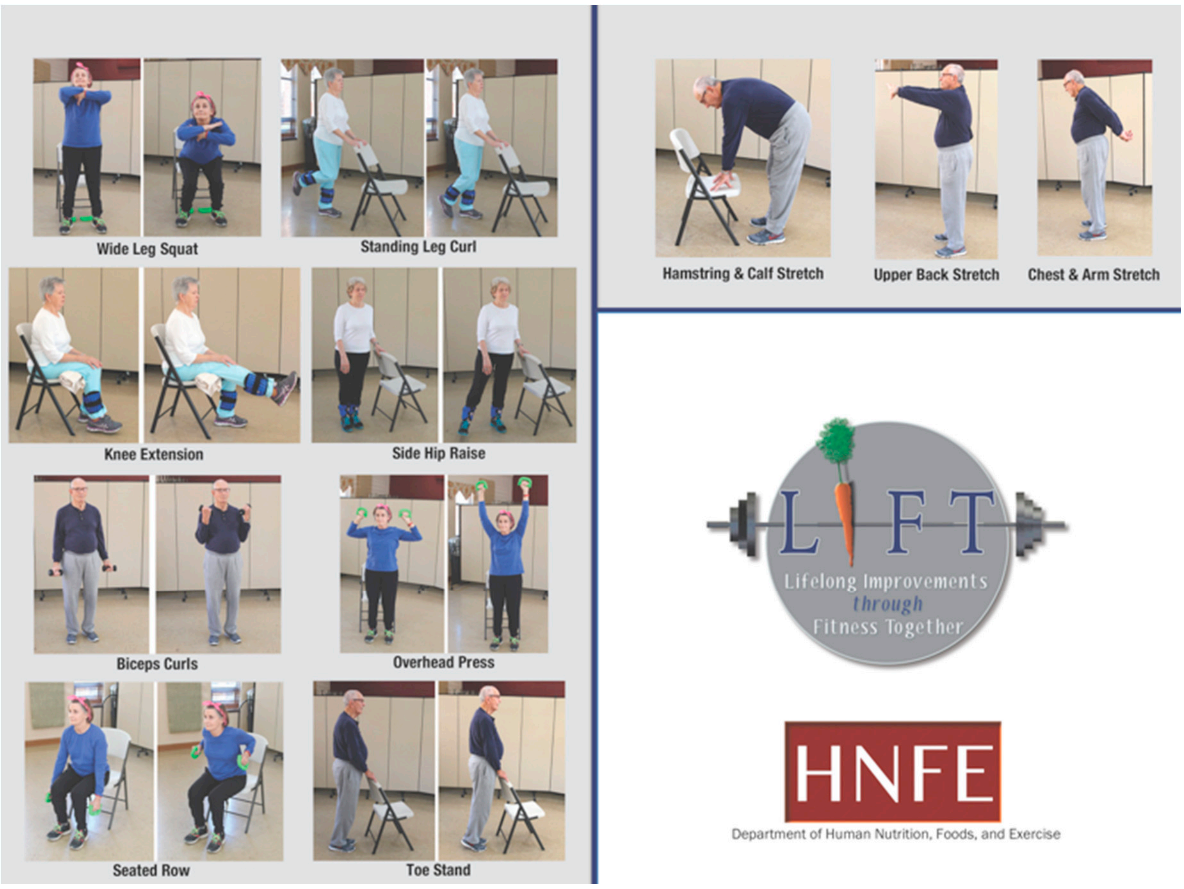

Supplementary Table 1: Session-by-session behavior change strategies and activities for LIFT

Session #	Session Objective	Targeted Principle	Why?	Suggested Group Activity
1	Introduction to program ***and*** group members.	-Interaction and communication	Everyone may or may not know each other. A game may act as an icebreaker and incorporates physical activity.	A. Active name game: With the group standing in a circle, have each person say their name and a corresponding exercise (e.g. Jumping Jack Jane). Everyone repeats the name and activity while doing the exercise and then the next person goes.<br>*If the group is too large, just have each individual introduce themselves with a corresponding exercise and then have the next person go (without everyone repeating and doing the exercise).*
2	Introduce group members, create a team name, develop phone tree.	- Role within the group<br>-Group distinctiveness/ team identity	Creating a phone tree encourages participates to communicate and support one another.<br><br>Team distinctiveness enables participants to feel a sense of belonging to the group.	A. Phone tree: Ask participants if they are comfortable sharing their name and best contact method with the group. Assign group members to call individuals if they miss more than two classes.<br><u>Team distinctiveness:</u><br>B: Establish a group name for the cohort (e.g. Aged to Perfection, Generation Fit, Portsmouth LIFTers).<br>C. Wear the same color t-shirts for class or community walks.
3	Collaborative group goal setting (physical activity completed outside of class).	-Group goals<br>-Group norms	Establishing a group goal sets a norm for class attendance and physical activity behaviors.<br><br>A group goal encourages team contribution and accountability more	A. Set a group goal. Examples:<br>1. 80% attendance for at least 14 of the 16 sessions.<br>2. 'Walk the state.' Any 15 minutes of aerobic activity outside of class counts as a mile to walk across the state.<br>3. Whoever gets the most exercise outside of class gets

			than an individual goal would.	______ (determine a nominal prize).
4	Discuss motivators of healthy lifestyle choices.	-Interaction and communication	Discussing personal motivators allows participants to learn about each other beyond surface level similarities and may enhance the positive group setting.	A. Discuss personal motivators for physical activity. Example prompts:<br>1. Discuss activities they remember doing as a youth.<br>2. Discuss activities, by the decade. What activity was your favorite in the 80s?<br>3. Talk about fruits and vegetables they enjoy growing or eating during certain seasons.
5	Develop plans for coping with physical activity barriers.	-Interaction and communication<br>-Problem solving	Participants may have the best intentions when it comes to being physically active, but barriers consistently pop up. Group discussion allows participants to share challenges and barriers to support each other in overcoming common barriers	A. Share methods for coping with barriers (e.g. such as establishing routines, getting to bed earlier, etc.).<br>B. List common barriers that people encounter.<br>C. Discuss previous methods used for overcoming barriers in the past.
6	Determine what resources are available for physical activity.	-Action planning	If membership fees, transportation, time, etc. are barriers for engaging in physical activity, identify free and convenient options for physical activity. This may help further alleviate barriers to meeting physical activity recommendations.	A. Open discussion: Discuss how participants could use community resources (parks, recreation centers, etc.) to meet physical activity recommendations.<br>B. List example exercises that can be done in community parks. (For example, use picnic tables for seated knee extensions and wide-leg squats or to provide balance for leg curls.)
7	Dietary behaviors influence physical activity	-Interaction and communication	Group discussion enhances a sense of 'belonging' among group members. The more opportunities they have to share	A. Ask participants to share favorite foods while exercising.<br>B. Provide examples of snacks that 'fuel' the body.<br>C. Distribute MyPlate for Older Adults (provided in manual).

	participation.		information about themselves, the more they will feel connected to those they are exercising with. This may happen organically, but as the instructor, you can provide prompts to ensure that even the more introverted participants have the opportunity to chat and contribute.	
8	Social integration and interaction outside of exercising.	-Group norms<br>-Interaction and communication	Offer opportunities for participants to be rewarded and acknowledged for their healthy behaviors (class attendance, continued progress with physical activity, positive attitudes, etc.).<br><br>Social gatherings outside of exercise enable participants to learn about each other.	A. Set up a potluck (for example, a midway point success potluck with healthy snacks).<br>*If participants are coming from work or have obligations after class, another option is for the instructor to provide a healthy snack for participants along with the recipe.*<br>B. Ask participants to bring a healthy recipe for a recipe exchange; discuss favorite healthy recipes during exercises. Or, ask participants to email their favorite healthy snack/meal recipes to the instructor; the instructor prints and brings them to next class.
9	Leadership roles to build confidence.	-Role within the group	Providing each participant with a role of 'leading' the group will help establish a sense of responsibility and accountability within the group. This may also help instructors identify people who may want to lead a class in the future.	A. Give participants the opportunity to 'lead' their group members through the exercises while counting out loud.<br>*Ask for volunteers and for others to count.*<br>B. Ask for at least one person to be the 'official counter' in class so that instructor can talk while doing the exercises.
10	Share successful behavior changes	-Interaction and communication	Encouraging small group interaction while exercising (with a partner) permits	A. Ask participants to work out with a partner in class. Each partner takes turns leading an exercise while the other counts

	for physical activity and fruit and vegetable consumption.	-Group size<br>-Feedback on goals<br>-Social support	members of the group to discuss and celebrate their improvements or find support where they still would like to make changes.	out loud.<br>B. Ask partners to share achievements and healthy lifestyle behavior changes made thus far.
11	Revisiting group and individual goal setting.	-Social support<br>-Interaction and communication<br>-Feedback on group goals<br>-Self monitoring<br>-Tailoring	Revisiting group and individual goals allows participants to analyze their progress and/or make adjustments to their goals where needed. Re-addressing goals before the end of the program will help prevent relapse to being inactive.	A. Ask for volunteers to share their individual goals and how they feel they have contributed towards the group goal.<br>B. Discuss any necessary changes to individual and group goals among the group.<br>C. Discuss if participants want to continue to meet as a group. If so, discuss where, how, who would lead, etc.
12	Strategies for maintaining long-term health behavior changes.	-Interaction and communication<br>-Self-monitoring	LIFT is about making lifelong changes—these topics and the opportunity for discussion can provide support in maintaining these changes.	A. Taking turns answering 'topic area' questions (voluntary and provided within manual) about motivators for physical activity, strategies to stay active, plans for staying physically active, etc. while exercising<br>B. Ask for volunteers to answer the questions and lead group exercises.
13	Motivators of long-term health behaviors.	-Interaction and communication<br>-Action planning<br>-Relapse prevention	Individuals are encouraged to focus on the positive outcomes associated with the physical activity they have completed thus far and determine how they will translate these habits into their daily routines.	Lead a group discussion from topics included:<br>A. What is different in their life/physical abilities now when compared to the start of class?<br>B. How will participants stay accountable for their own healthy lifestyle choices (e.g. physical activity and fruit and vegetable consumption)?<br>C. How will participants help each other stay accountable

				for their PA (e.g. continued use of phone tree)?
14	Establishing long-term coping and action plans.	-Interaction and communication<br>-Action planning	Participants may want to remain physically active at the end of LIFT. Provide opportunities for participants to schedule time outside of LIFT to meet and remain physically active.	A. Ask for volunteers to discuss opportunities to meet for physical activity outside of LIFT. How will they help each other stay motivated and on track to accomplish goals?<br>B. Invite the group to discuss what they need from you as the instructor or what opportunities they are curious about in the community.<br>C. As the instructor, set up facility tours. Talk with local facilities about costs, classes, and opportunities for aging adults in the community.
15	Recognizing group member contribution.	-Group roles<br>-Social support<br>-Feedback	Urge participants to acknowledge and celebrate their team members (outside of group leader providing the encouragement).	A. Ask for volunteers to express who in their group was most enthusiastic, most encouraging, etc. It can be more than one person or everyone in the group.<br>B. Go around in a circle and have each member say one positive attribute about the person to their right or how they've positively contributed to the team.
16	Acknowledge completion of group and individual goals.	-Feedback on group and individual goals<br>-Group norms	Having an 'end of program celebration' encourages conversations outside of exercise. This helps build relationships that may motivate them to continue physical activity after LIFT.	A. Set up a potluck style social to celebrate accomplishments.<br>B. As part of the celebration, if possible, provide some small incentives or prizes. Print out completion certificates for everyone.<br>C. Ask participants to email recipes to instructors so that recipes or a 'LIFT cookbook' can be provided to everyone at the last class.

Table S2. Physical activity and fruit and vegetable tracking sheet

Name: ____________________________

Directions

Record your minutes (Mins) of physical activity and your Cups of fruits and veggies completed outside of LIFT

**Day**	**Date:**		**Date:**		**Date:**		**Date:**		**Date:**		**Date:**		**Date:**		**Date:**	
	**Mins**	**Cups**	**Mins**	**Cups**	**Mins**	**Cups**	**Mins**	**Cups**	**Mins**	**Cups**	**Mins**	**Cups**	**Mins**	**Cups**	**Mins**	**Cups**
Sunday																
Monday																
Tuesday																
Wednesday																
Thursday																
Friday																
Saturday																
**Weekly Totals**																

Daily Minutes (Mins) of physical activity recommended for adults is at least 2 miles (30 mins. moderate intensity) on at least 5 days each week.

Daily Cups of fruits and vegetables recommended for active adults is about 5 cups every day.
